# Phylogenomic analysis of *Paenibacillus larvae* isolates from Slovakia predicts regional genomic clustering

**DOI:** 10.3389/fvets.2026.1872838

**Published:** 2026-07-08

**Authors:** Anna Kopcakova, Jana Kiskova, Silvia Ivorova, M. Pilar Garcillán-Barcia, Peter Pristas

**Affiliations:** 1 Laboratory of Microbial Genetics, Department of Digestive Tract Physiology, Institute of Animal Physiology, Centre of Biosciences of the Slovak Academy of Sciences, Kosice, Slovakia; 2 Department of Microbiology, Institute of Biology and Ecology, Faculty of Science, Pavol Jozef Safarik University in Kosice, Kosice, Slovakia; 3 Department of Animal Physiology, Institute of Biology and Ecology, Faculty of Science, Pavol Jozef Safarik University in Kosice, Kosice, Slovakia; 4 Instituto de Biomedicina y Biotecnología de Cantabria (IBBTEC, Consejo Superior de Investigaciones Científicas – Universidad de Cantabria), Santander, Cantabria, Spain

**Keywords:** American foulbrood, comparative genomic, diversity, honeybees, *Paenibacillus larvae*, phylogenomic analysis, sequencing, WGS

## Abstract

The *Paenibacillus larvae* is known as the causative agent of American foulbrood, which is widespread throughout the world. It is a highly contagious, fatal disease of honeybees. The aim of this study was to investigate the phylogenomic relationships of ERIC II genotype *P. larvae* isolates from Slovakia and to compare them with ERIC II genotype isolates originating from other countries. Phylogenomic analyses of *P. larvae* D3, 5S, and M1 isolates from Slovakia revealed a very low level of genetic diversity among our isolates, with low genome size variability (3.56 ± 0.04 Mbp) and average nucleotide and amino acid identity levels above 99%. Genomes showed similar gene counts (3,700 ± 59 genes per genome), with the highest variability in the mobile elements, especially bacteriophage-related genes (from 220 to 281 genes per genome). Comparative genomic analyses of several ERIC II and ERIC I types of *P. larvae* genomes available in the GenBank showed a clear geographical pattern, indicating that *P. larvae* strains spreading in the Slovak region differ from strains found in other parts of Europe and the rest of the world, thus indicating the possible existence of regionalism in the *P. larvae* species distribution.

## 1 Introduction

Accurate identification and typing of microorganisms are important steps not only for understanding microbial biogeography and heterogeneity but also for gaining knowledge about the pathogenicity of infectious agents and the epidemiology of microbial diseases. American Foulbrood Disease (AFB) is currently the most invasive bacterial disease affecting bee colonies worldwide. The disease is caused by the Gram-positive spore-forming bacterium *Paenibacillus larvae*, which causes major economic losses to the beekeeping industry worldwide each year by reducing bee populations, honey production, and other bee products. Because honeybees represent both an agricultural asset and an essential component of ecosystem health, AFB is of major veterinary and economic concern and has become one of the best-studied diseases of honeybees (1, 2). Even though this disease is called American Foulbrood, the origin of the disease is unknown. Outbreaks of AFB are most often caused by clonally related bacteria that share inherited biochemical and genetic properties, but sufficient diversity remains at the species level, leading to variations in the virulence of *P. larvae*. Despite its global distribution, the evolutionary origin and population structure of *P. larvae* remain incompletely resolved. Several molecular typing approaches have been applied to characterize *P. larvae* diversity. Enterobacterial repetitive intergenic consensus (ERIC)-PCR has been widely used for genotyping. ERIC-PCR generates genomic fingerprints based on amplification of repetitive intergenic elements but provides limited genome-wide resolution. Recently, five enterobacterial repetitive intergenic consensus (ERIC) genotypes (I-V) have been described (3). Differences in virulence between ERIC genotypes are associated with variation in disease progression, larval mortality rates, and outbreak severity (3, 4) highlighting the need for precise genotyping in veterinary diagnostics and surveillance. Genotype II is considered the most virulent (6). In recent years, the Multi Locus Sequence Typing (MLST) approach has been used for typing *P. larvae* isolates (7). It provides improved resolution but still interrogates only a small fraction of the genome: seven housekeeping genes. Proteomic approaches such as Matrix Assisted Laser Desorption Ionization Time of Flight Mass Spectrometry (MALDI-TOF MS) of total cellular proteins, which are combined with 16S rRNA sequencing, have been increasingly used as an alternative and fast tool for identifying, typing, and differentiating closely related strains (8, 9).

Our results so far indicate that MALDI-TOF MS is effective in differentiating *P. larvae*, and that 16S rRNA analysis provides a very low level of differentiation between *P. larvae* isolates and is unable to explain the observed phenotypic variability (6, 10). Understanding the intraspecies diversity of *P. larvae* is crucial for research into the epidemiology of the disease. Whole-genome sequencing (WGS) provides a powerful tool for veterinary epidemiology, enabling precise strain discrimination, identification of virulence-associated genes, and tracking of transmission pathways (11). Understanding genome-level variation is essential for assessing outbreak sources, monitoring pathogen spread, and improving disease control strategies.

In this study, we obtained drafts of whole-genome sequences of Slovak isolates and performed phylogenomic and comparative genomic analyses to evaluate genome-wide similarity, gene content variation, and the contribution of mobile genetic elements to strain diversification of closely related strains. By resolving intraspecific diversity at high genomic resolution, this study aims to improve understanding of AFB epidemiology in Slovakia and to provide genomic data relevant for veterinary surveillance and disease control programs.

## 2 Materials and methods

### 2.1 Origin of samples and identification of bacterial isolates

Bacterial isolates of *P. larvae* D3, 5S, and M1 were obtained from the collection of microorganisms of the Centre of Biosciences of the Slovak Academy of Sciences, Institute of Animal Physiology in Kosice. The isolates were isolated from different localities in Eastern Slovakia from infected brood combs (D3 – Velka Lodina; 5S – Kosice and M1 – Lorincik) and identified and typed by using a combination of 16S rRNA sequencing, MALDI-TOF MS spectrometry, and ERIC-PCR (see publication (6)).

### 2.2 Isolation of DNA, sequencing, and phylogenetic analysis

The DNA was extracted from bacterial cultures grown in MYPGP medium (1.5% yeast extract, 0.3% K2PO4, 0.2% glucose, 0.1% sodium pyruvate, and 1.0% Mueller–Hinton broth) using the GenElute™ Bacterial Genomic DNA Kit according to the manufacturer’s protocol (Sigma Aldrich, St. Louis, United States). The quality and quantity of the extracted DNA were confirmed by agarose electrophoresis in 0.8% agarose gel in a TAE buffer and by using NanoDrop 2000c Spectrophotometer (Thermo Scientific, United States) and subsequently subjected to whole-genome sequencing by Eurofins Genomics Europe Pharma and Diagnostics (Constance, Germany), using Illumina technology, mode NovaSeq PE 150. The quality of raw sequences was checked using FastQC v 0.11.9 (12). Trimmomatic v0.39 (13) was used to remove sequences with a quality score lower than Q20. The high-quality sequences were *de novo* assembled using the SPAdes tool (14). Finally, the contigs shorter than 200 bp were excluded from the obtained draft genomes. Draft genome sequences were deposited in the GenBank database^1^ under the following accession numbers: JBLOBJ000000000 and GCA_047786425.1 for the D3 isolate, JBLOBH000000000 and GCA_047786405.1 for the 5S isolate, and JBLOBI000000000 and GCA_047786385.1 for the M1 isolate. All quality metrics and basic genome descriptions are shown in Table 1.

**Table 1:** The features of *P. larvae* genomes originating from Slovakia according to the RAST server.

The features of genomes	D3 isolate	5S isolate	M1 isolate
	JBLOBJ000000000	JBLOBH000000000	JBLOBI000000000
Genome size (bp)	3,714,613	3,708,420	3,679,277
Sequenced reads	6,396,195	7,601,786	8,891,576
Trimmed reads	6,395,799	7,601,503	8,891,243
Genome coverage	436x	518x	606x
GC content (%)	44.6	44.6	44.6
Largest contig (bp)	100,864	100,865	101,339
N50 (bp)	25,137	23,599	25,134
L50	45	47	45
Number of contigs	359	381	358
Completeness (%)*	99.19	99.19	99.19
Contamination (%)	0.02	0	0

* Genome completeness and contamination calculated by the CheckM tool using the NCBI database.

Phylogenetic position of the studied isolates within *P. larvae* strains of known ERIC genotype (genome assemblies are available in the GenBank database with accession numbers GCA_000511405.1, GCA_002951895.1, GCA_002082155.1, GCA_032710345.1, GCA_002003265.1, GCA_002951875.1, and known genotype) was performed using digital DNA–DNA hybridization (dDDH) and construction of a Genome BLAST Distance Phylogeny (GBDP) phylogenetic tree using the Type (Strain) Genome Server (TYGS) tool (15). The analysis was supplemented by calculating the Average Amino Acid Identity (AAI) and Average Nucleotide Identity (ANI) values using the EzAAI tool (16) and the JspeciesWS server (17).

Annotation of the studied genomes was performed using the Rapid Annotation using Subsystem Technology (RAST) (18). The PhageBoost server^2^ was used to detect prophage regions (at least 7 bacteriophage related genes in a row) (19). The presence of plasmids in draft sequences was analyzed using an online search against the PLSDB database (available at https://ccb-microbe.cs.uni-saarland.de/plsdb2025/) (20).

## 3 Results

In this study, the draft whole-genome sequences were analyzed for three ERIC II genotype *P. larvae* isolates (D3, 5S, and M1) obtained from three localities in Eastern Slovakia for performing phylogenomic and comparative genomic analyses to evaluate genome-wide similarity, gene content variation, and the contribution of mobile genetic elements to strain diversification of closely related strains. The features of genomes are described in Table 1.

**Figure 1:**
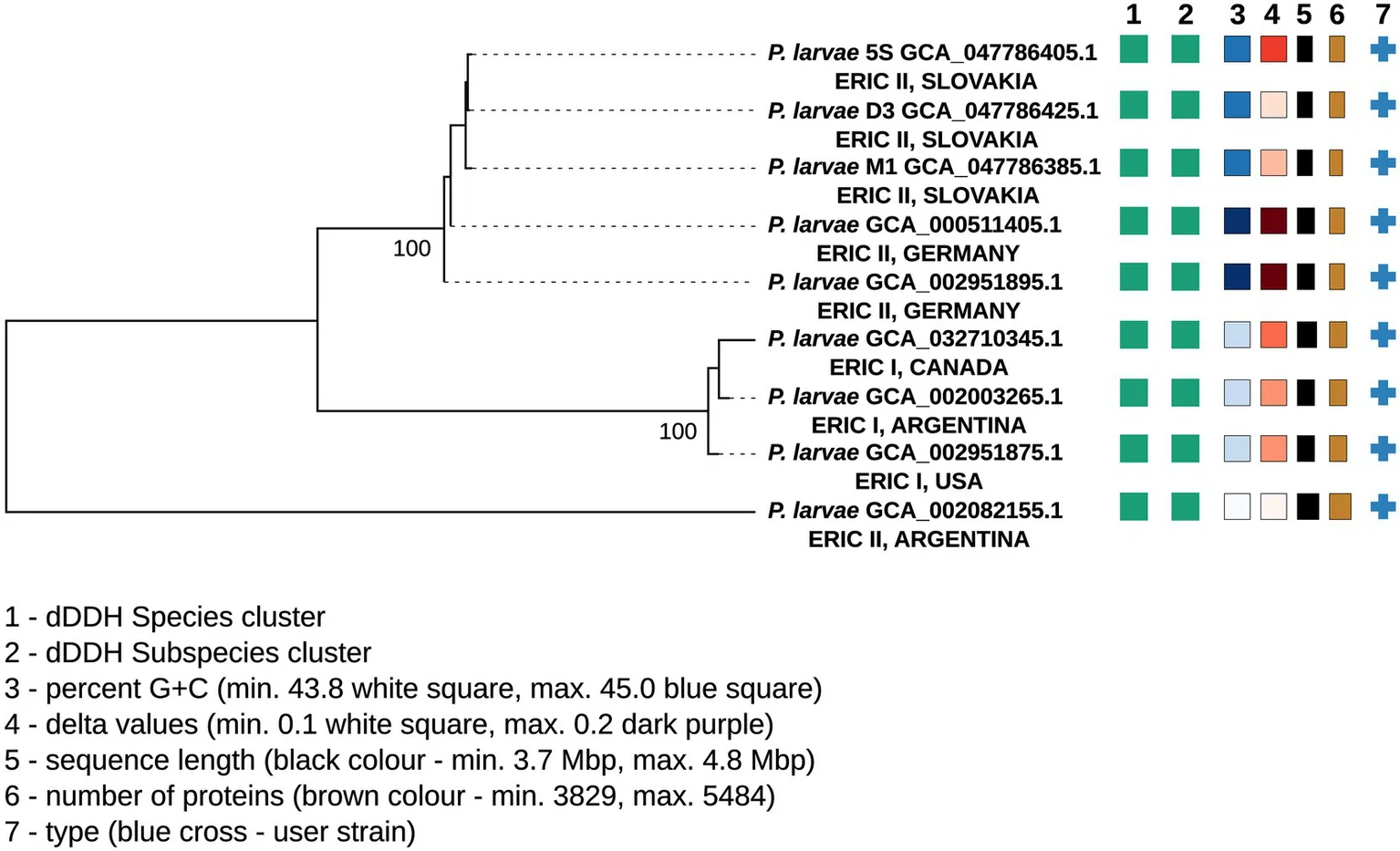
TYGS result for the *Paenibacillus* data set. Tree inferred with FastME 2.1.6.1 from GBDP distances calculated from genome sequences. Branch lengths are scaled in terms of the GBDP distance formula d5; numbers under branches are GBDP pseudo-bootstrap support values from 100 replications. Reproduced from the Type (Strain) Genome Server, https://tygs.dsmz.de, licensed under CC BY-SA 4.0.

Phylogenomic analyses of three genomes indicated very low genome size variability (3.71 ± 0.03 Mbp) among isolates from Slovakia. The analysis by ANI, AAI, and dDDH showed very high genetic similarity values exceeding 99%. The lower AAI, ANI, and dDDH values were observed for the genomes from other areas of the world (see Table 2). The isolates from Slovakia were placed in a single clade in the GBDP tree constructed using the TYGS server (25–27), and together with isolates from Germany formed a well-separated clade (Figure 1).

**Table 2:** The phylogenomic analysis of *P. larvae* genomes originating from Slovakia and different countries worldwide according to ANI, AAI, and dDDH comparison.

Genomes of *P. larvae*	Country	Genotype	ANI in %			AAI in %			dDDH (d4 in %)		
			5S	M1	D3	5S	M1	D3	5S	M1	D3
5S GCA_047786405.1	Slovakia	ERIC II	–	99.87	99.96	–	99.95	99.93	–	99.9	99.9
M1 GCA_047786385.1	Slovakia	ERIC II	99.87	–	99.89	99.95	–	99.98	99.9	–	100.0
D3 GCA_047786425.1	Slovakia	ERIC II	99.96	99.89	–	99.93	99.98	–	99.9	100.0	–
GCA_000511405.1	Germany	ERIC II	99.96	99.90	99.95	99.97	99.98	99.98	99.9	99.7	99.9
GCA_002951895.1	Germany	ERIC II	99.96	99.89	99.96	99.97	99.98	99.98	99.9	99.7	99.9
GCA_002082155.1	Argentina	ERIC II	98.22	98.13	98.20	98.63	98.63	98.62	90.6	90.4	90.5
GCA_032710345.1	Canada	ERIC I	99.10	99.06	99.09	99.28	99.29	99.28	96.2	96.0	96.1
GCA_002003265.1	Argentina	ERIC I	99.19	99.16	99.19	99.26	99.28	99.27	96.5	96.2	96.4
GCA_002951875.1	USA	ERIC I	99.22	99.17	99.21	99.31	99.31	99.31	96.5	96.3	96.4

The D3, 5S, and M1 genomes exhibited very small differences in gene counts (3,700 ± 59 genes per genome) predicted using the RAST server (18), with the highest variability observed in mobile genetic elements, particularly in the genes of putative bacteriophage origin (ranging from 220 to 281 genes per genome, Table 3). Similarly, the PhageBoost server predicted 194 to 258 genes per genome in our genomes, whereas in the genome DSM25430, it was 338 per genome (data not shown). Different sets of plasmids were detected in the genomes of D3, 5S, and M1 isolates during search against the PLSDB database (20) using a stringent search (minimal identity 0.99). In the genomes of D3 and M1 isolates, 3 plasmids were detected (the same set as in the genome of the geographically related DSM 25430 strain from Germany). In the 5S genome, a single plasmid (NZ_CP019653.1, *P. larvae* subsp. *larvae* DSM 25430 plasmid unnamed1, Table 3) was detected, indicating that mobile gene elements contribute to the observed genomic variability in *P. larvae.*

**Table 3:** The comparative genomics of *P. larvae* isolates from Slovakia and the closest ERICII type relative from Germany.

	DSM25430 strain	D3 isolate	5S isolate	M1 isolate
	NZ_CP019652	JBLOBJ000000000	JBLOBH000000000	JBLOBI000000000
	Germany	Slovakia	Slovakia	Slovakia
Genome size (bp)	4,056,006	3,714,613	3,708,420	3,679,277
Number of				
Coding sequences	3,796	4,401	4,393	4,369
RNAs	102	77	76	77
Putative prophage regions	20	11	10	8
Phage-related genes	422	255	220	281
The presence of a plasmid				
pPLA2_10 (NC_023147.1)	+	+	−	+
Unnamed 1 (NZ_CP019653.1)	+	+	−	+
Unnamed 2 (NZ_CP019654.1)	+	+	+	+
Number of genes in category				
Virulence, disease and defense	63	67	66	62
Stress response	77	80	79	79
Regulation and cell signaling	53	58	58	46
DNA metabolism	343	124	122	121
Motility and chemotaxis	38	73	73	73

Genomic comparison with the DSM25430 genome showed that genomes of *P. larvae* isolates from Slovakia show a similar number of genes in most categories, including the Virulence, Disease and Defense, Stress Response, and Regulation and Cell Signaling categories identified by RAST annotation (18), but show a significantly lower number of genes for RNA and in the DNA Metabolism and Motility and Chemotaxis categories (Table 3). In the DNA Metabolism category, all Slovak isolates lack multiple genes participating in DNA repair.

## 4 Discussion

AFB represents one of the most destructive bacterial diseases affecting honeybee (*Apis mellifera*) colonies worldwide, with significant ecological and economic impacts. The etiological agent of the disease, *P. larvae*, exhibits considerable genetic diversity, which has been linked to differences in virulence, epidemiology, and transmission dynamics. In Slovakia and other countries worldwide, despite long-term monitoring and control measures, AFB remains a persistent threat, particularly in regions with high colony density and frequent movement of beekeeping materials. Understanding the routes of AFB spread and the population structure of *P. larvae* is therefore essential for improving disease management strategies. Traditional genotyping methods, such as ERIC-PCR, have identified several genotypes, among which ERIC II is often associated with increased virulence and rapid disease progression. However, these approaches provide only limited resolution for elucidating fine-scale genetic relationships and transmission pathways. In this context, the WGS combined with phylogenomic and comparative genomic analyses offers a powerful framework for high-resolution characterization of pathogen populations (5).

For a better understanding of the American foulbrood spread routes and variability of *P. larvae*, the etiological agent of AFB in Slovakia, a phylogenomic approach and comparative genomics were used in this study. Draft whole-genome sequences were obtained for three ERIC II genotype *P. larvae* isolates (D3, 5S, and M1) obtained from three localities in Eastern Slovakia separated by more than 15 km, thereby minimizing the probability of direct epidemiological linkage. This spatial distribution provides an opportunity to assess genomic variability within the ERIC II lineage at a regional scale and to explore potential patterns of dissemination independent of immediate local transmission. Phylogenomic analyses predicted a very low genome size variability (3.71 ± 0.03 Mbp) in isolates from Slovakia, which is the lowest among *P. larvae* genomes (of about 4 Mbp for the isolates in the GenBank database) available in the GenBank database. Three Slovak origin genomes showed very high genetic similarity, with ANI, AAI, and dDDH values exceeding 99%. The distance between the localities is greater than the usual flight range of worker bees, so robbing or bee drifting cannot explain the similarities observed. Similar low genetic variability was observed in *P. larvae* outbreaks in Slovenia (21), where the ERIC II genotype was detected, or in Australia, where the ERIC I genotype was confirmed (22). On the other hand, lower AAI, ANI, and dDDH values (see Table 2) were observed for the genomes from other areas of the world (see Figure 1), and there is a correlation between genomic and geographic distances between isolates. Isolates from Slovakia were placed in a well-separated branch in the GBDP tree, indicating that the locally differentiated genomic lineage ERIC II of *P. larvae* spreads on the territory of Eastern Slovakia, distinct from ERIC II and ERIC I genomic lineages from Germany or North and South America (Figure 1). The D3, 5S, and M1 genomes exhibited very small differences in gene counts (3,700 ± 59 genes per genome) detected using the RAST server (18), with the highest variability observed in mobile genetic elements, particularly in the genes of putative bacteriophage origin (ranging from 220 to 281 genes per genome, Table 3). Confirming the presence of prophages in our genomes was realized by adding analysis using the PhageBoost server (see footnote 2). Analysis predicted 194 to 258 genes per genome, and in the genome DSM25430, it was 338 per genome (data not shown). In *P. larvae*, a large part of the genome size variability is due to integrated bacteriophages (prophages). Comparing our genomes with the German genome DSM25430 showed a difference in the number of phage regions /genes. Our genomes had approximately half the number of phage regions / genes compared to the DSM25430 genome (see Table 3). This means that if our strains have lost or never acquired prophages, their genomes may be up to hundreds of kilobases smaller. Different sets of plasmids were detected in the genomes of D3, 5S, and M1 isolates, too, using the PLSDB database (20), which indicates that mobile gene elements contribute to the observed genomic variability in *P. larvae.* Mobile genetic elements are DNA segments capable of moving between genomes or within a genome. In the disease of American foulbrood caused by the bacterium *P. larvae*, they play a very important role. They influence the virulence of the bacterium, its ability to adapt, horizontal gene transfer, and, last but not least, the epidemiology of the disease in bee populations. Plasmids and transposons can carry resistance genes and promote the selection of resistant strains. Although antibiotic therapy is mostly banned in Europe, selection pressure may have affected *P. larvae* populations in the past. Mobile gene elements accelerate recombination, genome rearrangement, and the creation of new variants. These factors, therefore, significantly complicate disease control, diagnosis, and, last but not least, virulence prediction (23, 24).

Next, genomic comparison with the German origin DSM25430 strain genome showed that genomes of *P. larvae* isolates from Slovakia show a similar number of genes in most categories, including the Virulence, Disease and Defense, Stress Response, and Regulation and Cell Signaling categories identified by RAST annotation, but show a significantly lower number of genes for RNA and in the DNA Metabolism and Motility and Chemotaxis categories (Table 3). In the DNA Metabolism category, all Slovak isolates lack multiple genes participating in DNA repair. On the other hand, in the Motility and Chemotaxis category, the German strain completely lacks genes in the Flagellum subcategory, where genes for flagellar biosynthesis, flagellar motor stator proteins, and flagellar motility belong. The biological importance of observed differences must be confirmed experimentally, but these findings point to distinct genomic adaptations in Slovak isolates and the reference strain, particularly in functions related to genome stability and motility, which may have implications for their pathogenicity, environmental persistence, and evolutionary relationships. Variability in metabolic functions may reflect differences in the ability of individual strains to utilize available nutrients or adaptation to specific environmental conditions. Differences in genes related to motility and chemotaxis may indicate differences in mechanisms of colonization and interaction with the host. The lower representation of genes classified into DNA and RNA categories may be related to variability in regulatory mechanisms, DNA repair processes, or genes involved in nucleic acid metabolism. Since the analyzed strains belonged to the same ERIC II genotype, it is likely that the observed differences represent a variable component of the genome and reflect microevolutionary processes occurring within this genetic lineage, while genes involved in virulence and basic cellular functions remain evolutionarily conserved.

Our findings predict a possible regionalism in the occurrence of *P. larvae* species, since Slovak isolates differ from previously reported strains from other regions of Europe and the world. The isolates from Germany form a neighboring branch to isolates of Slovakia, even though they differ significantly in genomic characteristics (e.g., genome size 4.1 Mbp versus 3.7 Mbp for Slovak isolates). On the other hand, the plasmid occurrence in 2 Slovak isolates is identical to the profile of the German isolate. The cause of the possible observed regionalism is not yet clear, but it could be the transmission of *P. larvae* through human activity. Human-mediated transmission is one of the most important epidemiological factors for American foulbrood, as natural spread of the disease is possible but usually geographically limited (up to 10 km). In contrast, humans can inadvertently transfer *P. larvae* spores over long distances and between many colonies in a short time. A key problem is the extraordinary resistance of the spores. The spores can survive in honey, wax, propolis, wooden parts of hives, or on tools for decades without losing infectivity. As a result, even old or apparently clean material can become a source of infection. Epidemiologically, it is important that very small amounts of spores are sufficient to cause larval infection. One of the most important mechanisms of spreading is the movement of bee colonies. Commercial nomadic bees for pollination or trade in brood and queens create extensive contact networks between regions. If a colony is infected subclinically, i.e., without obvious visible symptoms, it can be relocated before the disease is diagnosed. Such “silent” spread significantly complicates disease control and promotes the geographical expansion of certain genotypes of *P. larvae.* Contaminated hive material is also of great importance. Frames, partitions, wax, feeders, or used hives can contain high concentrations of spores. Recycling wax is particularly risky, as spores can survive normal processing conditions. Similarly, honey used to feed bees can be a source of infection if it comes from contaminated colonies. All of the above factors are significant epidemiological mediators of this invasive bacterial disease.

## 5 Conclusion

This study provides new genomic data for *P. larvae* isolates from Europe and highlights the need for broader genomic sampling to clarify transmission routes and population structure. The aim of the future research is to obtain and analyze additional data (genomes) and confirm (or exclude) the cause of transmission and virulence of *P. larvae* associated with the presence of mobile genetic elements, including plasmids, transposons, and bacteriophages, which have been identified in Slovak *P. larvae* genomes. These elements may play a critical role in shaping bacterial virulence, adaptability, and evolutionary trajectories. However, their functional significance in the context of *P. larvae* pathogenicity and transmission remains to be conclusively determined. Future studies integrating genomic, functional, and epidemiological data will be essential to confirm or exclude their involvement in virulence modulation and disease spread.

A better understanding of these mechanisms could ultimately contribute to the development of more effective surveillance, prevention, and control strategies targeting AFB. Considering the significant economic and ecological impact of AFB on apiculture, such advancements are crucial not only for Slovakia but also for the global beekeeping community.

## Data Availability

The datasets presented in this study can be found in online repositories. The names of the repository/repositories and accession number(s) can be found at: https://www.ncbi.nlm.nih.gov/genbank/, JBLOBJ000000000; https://www.ncbi.nlm.nih.gov/genbank/, JBLOBH000000000; https://www.ncbi.nlm.nih.gov/genbank/, JBLOBI000000000.

## Footnotes

1
https://www.ncbi.nlm.nih.gov/genbank

2
https://phageboost.ku.dk/

## References

[1] Manhong Y Xiaoyuan L Fengping Y Bin Z. Beneficial bacteria as biocontrol agents for American foulbrood disease in honey bees (*Apis mellifera*). J Insect Sci. (2023) 23:6. doi: 10.1093/jisesa/iead013, 36947033 PMC10032306

[2] Okamoto M Furuya H Sugimoto I Kusumoto M Takamatsu D. A novel multiplex PCR assay to detect and distinguish between different types of *Paenibacillus larvae* and *Melissococcus plutonius*, and a survey of foulbrood pathogen contamination in Japanese honey. J Vet Med Sci. (2022) 84:390–9. doi: 10.1292/jvms.21-0629, 35082220 PMC8983297

[3] Beims H Bunk B Erler S Hohr KI Spröer C Pradella S . Discovery of *Paenibacillus larvae* ERIC V: phenotypic and genomic comparison to genotypes ERIC I-IV reveal different inventories of virulence factors which correlate with epidemiological prevalences of American foulbrood. Int J Med Microbiol. (2020) 310:151394. doi: 10.1016/j.ijmm.2020.151394, 31959580

[4] Genersch E Forsgren E Pentikäinen J Ashiralieva A Rauch S Kilwinski J . Reclassification of *Paenibacillus larvae* subsp. *pulvifaciens* and *Paenibacillus larvae* subsp. *larvae* as *Paenibacillus larvae* without subspecies differentiation. Int J Syst Evol Microbiol. (2006) 56:501–11. doi: 10.1099/ijs.0.63928-0, 16514018

[5] Kumar S Suvidhi Choudhary A. The impact of genomic sequencing on veterinary diagnostics. J Ethol Anim Sci. (2024) 6. doi: 10.23880/jeasc-16000137

[6] Kopcakova A Salamunova S Javorsky P Sabo R Legath J Ivorova S . The application of MALDI-TOF MS for a variability study of *Paenibacillus larvae*. Vet Sci. (2022) 9:521. doi: 10.3390/vetsci9100521, 36288134 PMC9610059

[7] Matiašovic J Bzdil J Papežíková I Čejková D Vasina E Bizos J . Genomic analysis of *Paenibacillus larvae* isolates from the Czech Republic and the neighbouring regions of Slovakia. Res Vet Sci. (2023) 158:34–40. doi: 10.1016/j.rvsc.2023.03.007, 36913910

[8] Cabrolier N Sauget M Bertrand X Hocquet D. Matrix-assisted laser desorption ionization–time of flight mass spectrometry identifies *Pseudomonas aeruginosa* high-risk clones. J Clin Microbiol. (2015) 53:1395–8. doi: 10.1128/jcm.00210-15, 25653397 PMC4365210

[9] De Carolis E Vella A Vaccaro L Torelli R Spanu T Fiori B . Application of MALDI-TOF mass spectrometry in clinical diagnostic microbiology. J Infect Dev Ctries. (2014) 8:1081–8. doi: 10.3855/jidc.3623, 25212071

[10] Lebano I Fracchetti F Vigni ML Mejia JF Felis G Lampis S. MALDI-TOF as a powerful tool for identifying and differentiating closely related microorganisms: the strange case of three reference strains of *Paenibacillus polymyxa*. Sci Rep. (2024) 14:2585. doi: 10.1038/s41598-023-50010-w, 38297004 PMC10831075

[11] Muhee A Pandit A Jan S Khan IS Hassan N Bhat RA . Whole genome sequencing reveals environmental pathogen misidentification and potential for cross-phylum antimicrobial resistance gene transfer in bovine mastitis: a pilot genomic study. BMC Vet Res. (2026) 22:134. doi: 10.1186/s12917-025-05280-z, 41535901 PMC12933902

[12] Andrews S. (2010) FastQC: a quality control tool for high throughput sequence data. Available online at: http://www.bioinformatics.babraham.ac.uk/projects/fastqc (Accessed June 27, 2025).

[13] Bolger AM Lohse M Usadel B. Trimmomatic: a flexible trimmer for Illumina sequence data. Bioinformatics. (2014) 30:2114–20. doi: 10.1093/bioinformatics/btu170, 24695404 PMC4103590

[14] Prjibelski A Antipov D Meleshko D Lapidus A Korobeynikov A. Using SPAdes de novo assembler. Curr Protoc Bioinformatics. (2020) 70:e102. doi: 10.1002/cpbi.102, 32559359

[15] Meier-Kolthoff JF Göker M. TYGS is an automated high-throughput platform for state-of-the-art genome-based taxonomy. Nat Commun. (2019) 10:2182. doi: 10.1038/s41467-019-10210-3, 31097708 PMC6522516

[16] Kim D Park S Chun J. Introducing EzAAI: a pipeline for high throughput calculations of prokaryotic average amino acid identity. J Microbiol. (2021) 59:476–80. doi: 10.1007/s12275-021-1154-0, 33907973

[17] Richter M Rosseló-Móra R. Shifting the genomic gold standard for the prokaryotic species definition. Proc Natl Acad Sci USA. (2009) 106:19126–31. doi: 10.1073/pnas.0906412106, 19855009 PMC2776425

[18] Aziz RK Bartels D Best AA Dejongh M Disz T Edwards RA . The RAST server: rapid annotations using subsystems technology. BMC Genomics. (2008) 9:75. doi: 10.1186/1471-2164-9-75, 18261238 PMC2265698

[19] Sirén K Millard A Petersen B Gilbert MTP Clokie MRJ Sicheritz-Pontén T. Rapid discovery of novel prophages using biological feature engineering and machine learning. NAR Genom Bioinform. (2021) 3:lqaa109. doi: 10.1093/nargab/lqaa109, 33575651 PMC7787355

[20] Schmartz GP Hartung A Hirsch P Kern F Fehlmann T Müller R . PLSDB: advancing a comprehensive database of bacterial plasmids. Nucleic Acids Res. (2021) 50:D273–8. doi: 10.1093/nar/gkab1111, 34850116 PMC8728149

[21] Papić B Diricks M Kušar D. Analysis of the global population structure of *Paenibacillus larvae* and outbreak investigation of American foulbrood using a stable wgMLST scheme. Front Vet Sci. (2021) 8:582677. doi: 10.3389/fvets.2021.582677, 33718463 PMC7952629

[22] Worden P Webster A Gandhi K Gupta R Deutscher AT Hornitzky M . Genomic diversity and tracing of *Paenibacillus larvae* in Australia: implications for American foulbrood outbreak surveillance in low-diversity populations. Microb Genom. (2025) 11:001374. doi: 10.1099/mgen.0.001374, 40327033 PMC12163730

[23] Kumavath R Gupta P Tatta ER Mohan MS Salim SA Busi S . Unraveling the role of mobile genetic elements in antibiotic resistance transmission and defense strategies in bacteria. Front. Syst. Biol. (2025) 5. doi: 10.3389/fsysb.2025.1557413 PMC12342005 40810119

[24] Djukic M Brzuszkiewicz E Fünfhaus A Voss J Gollnow K Poppinga L . How to Kill the Honey Bee Larva: Genomic Potential and Virulence Mechanisms of *Paenibacillus larvae*. PLOS One. (2014). doi: 10.1371/journal.pone.0090914 PMC3944939 24599066

[25] Lefort V Desper R Gascuel O. FastME 2.0: A comprehensive, accurate, and fast distance-based phylogeny inference program. Mol Biol Evol. (2015) 32: 2798–2800. doi: 10.1093/molbev/msv150 PMC4576710 26130081

[26] Farris JS. Estimating phylogenetic trees from distance matrices. Am Nat. (1972) 106: 645–667. doi: 10.1086/282802

[27] Kreft L Botzki A Coppens F Vandepoele K Van Bel M. PhyD3: A phylogenetic tree viewer with extended phyloXML support for functional genomics data visualization. Bioinformatics. (2017). 33: 2946–2947. doi: 10.1093/bioinformatics/btx324 28525531

