# Correction: Phylogenomic analysis of *Paenibacillus larvae* isolates from Slovakia predicts regional genomic clustering

**DOI:** 10.3389/fvets.2026.1946430

**Published:** 2026-09-17

**Authors:** Anna Kopcakova, Jana Kiskova, Silvia Ivorova, M. Pilar Garcillán-Barcia, Peter Pristas

**Affiliations:** 1 Laboratory of Microbial Genetics, Department of Digestive Tract Physiology, Institute of Animal Physiology, Centre of Biosciences of the Slovak Academy of Sciences, Kosice, Slovakia; 2 Department of Microbiology, Institute of Biology and Ecology, Faculty of Science, Pavol Jozef Safarik University in Kosice, Kosice, Slovakia; 3 Department of Animal Physiology, Institute of Biology and Ecology, Faculty of Science, Pavol Jozef Safarik University in Kosice, Kosice, Slovakia; 4 Instituto de Biomedicina y Biotecnología de Cantabria (IBBTEC, Consejo Superior de Investigaciones Científicas – Universidad de Cantabria), Santander, Cantabria, Spain

**Keywords:** American foulbrood, comparative genomic, diversity, honeybees, *Paenibacillus larvae*, phylogenomic analysis, sequencing, WGS

Affiliation “Department of Microbiology, Institute of Biology and Ecology, Faculty of Science, Pavol Jozef Safarik University in Kosice, Kosice, Slovakia” was omitted from the paper. This affiliation has now been added for authors Jana Kiskova and Peter Pristas.

Affiliation 2 “Department of Animal Physiology, Faculty of Natural Science, Institute of Biology and Ecology, Pavol Jozef Safarik University, Kosice, Slovakia” was erroneously included in the paper. This affiliation has now been removed for authors Jana Kiskova and Peter Pristas.

In the published article, the wording and structure of the following affiliations were incorrect:

- Affiliation 1 was published as “Laboratory of Microbial Genetics, Department of Digestive Tract Physiology, Centre of Biosciences of the Slovak Academy of Sciences, Institute of Animal Physiology, Kosice, Slovakia”. The correct affiliation is “Laboratory of Microbial Genetics, Department of Digestive Tract Physiology, Institute of Animal Physiology, Centre of Biosciences of the Slovak Academy of Sciences, Kosice, Slovakia”.
- Affiliation 2 was published as “Department of Animal Physiology, Faculty of Natural Science, Institute of Biology and Ecology, Pavol Jozef Safarik University, Kosice, Slovakia”. The correct affiliation is now Affiliation 3: “Department of Animal Physiology, Institute of Biology and Ecology, Faculty of Science, Pavol Jozef Safarik University in Kosice, Kosice, Slovakia”.

There were several mistakes in Table 3 as published:

- In row 3, the text “genes in category” was erroneously included. The correct sub-heading is “Number of”.
- In row 8, “The presence of a plasmid” should have been formatted as a sub-heading.
- Between the row for Unnamed 2 (NZ_CP019654.1) and the row for Virulence, disease and defense, a sub-heading labeled “Number of genes in category” was omitted.

The corrected version of Table 3 and its caption appear below.

**Table 3:** The comparative genomics of *Paenibacillus larvae* isolates from Slovakia and the closest ERICII type relative from Germany.

	DSM25430 strain	D3 isolate	5S isolate	M1 isolate
	NZ_CP019652	JBLOBJ000000000	JBLOBH000000000	JBLOBI000000000
	Germany	Slovakia	Slovakia	Slovakia
Genome size (bp)	4,056,006	3,714,613	3,708,420	3,679,277
Number of				
Coding sequences	3,796	4,401	4,393	4,369
RNAs	102	77	76	77
Putative prophage regions	20	11	10	8
Phage-related genes	422	255	220	281
The presence of a plasmid				
pPLA2_10 (NC_023147.1)	+	+	-	+
Unnamed 1 (NZ_CP019653.1)	+	+	-	+
Unnamed 2 (NZ_CP019654.1)	+	+	+	+
Number of genes in category				
Virulence, disease and defense	63	67	66	62
Stress response	77	80	79	79
Regulation and cell signaling	53	58	58	46
DNA metabolism	343	124	122	121
Motility and chemotaxis	38	73	73	73

The original version of this article has been updated.

